# Melanoma Cells Exposed to Clomiphene Citrate Respond With Cell Cycle Arrest and Reduced Invasiveness

**DOI:** 10.1002/jat.70198

**Published:** 2026-04-09

**Authors:** Thaís S. Ribeiro, Felipe H. S. Silva, Ana Paula G. S. Miranda, Isabella T. Borges, Pedro H. D. M. Prazeres, Marcia C. F. Ferreira, Enio Ferreira, Fernando M. Reis, Helen L. Del Puerto

**Affiliations:** ^1^ Department of General Pathology, Institute of Biological Sciences Federal University of Minas Gerais Belo Horizonte Brazil; ^2^ Department of Obstetrics and Gynecology Federal University of Minas Gerais Belo Horizonte Brazil

**Keywords:** BAX, cadherins, clomiphene citrate, melanoma, ovulation induction, SOD2

## Abstract

Epidemiological studies have suggested that clomiphene citrate (CC) exposure may increase the risk of malignant melanoma, but mechanisms are unknown. We investigated the effects of CC on melanoma cell line A375. Cells were treated with CC at 2, 20, 200, and 2000 ng/mL, followed by assessment of cell viability and metabolism, cell cycle, migration, and expression of genes associated with epithelial–mesenchymal transition and mitochondrial oxidative stress. At 48 h, 2000‐ng/mL CC reduced cell viability by 80% (*p* < 0.0001), induced early G1‐phase arrest (*p* < 0.0001), and impaired cell migration. Gene expression analysis revealed decreased N‐cadherin/*CDH2* (*p* < 0.05) and *SOD2* (*p* < 0.01) gene expression in the presence of CC, suggesting antimetastatic and prooxidant effects. These results indicate that direct exposure to CC does not promote aggressive melanoma cell behavior in vitro.

## Abstract 2

Clomiphene citrate treatment does not increase the malignant potential of human melanoma cells in vitro but may even decrease some of their malignant features.

## 1 Introduction

Epidemiological studies have investigated whether exposure to fertility treatments that induce supraphysiological estrogen levels could increase the long‐term risk of melanoma. The available evidence suggests either a slight increase in melanoma risk among women treated with clomiphene citrate (CC) (Brinton et al. 2015; Calderon‐Margalit et al. 2009) or no association (Althuis et al. 2005; Hannibal et al. 2008). Due to the many limitations of observational studies in establishing causal relationships between drug exposure and subsequent disease development, the putative association between CC and melanoma risk remains speculative.

Proposed mechanisms underlying this association include a temporary increase in serum estrogen levels, by analogy with pregnancy and oral contraceptive use, which have been suggested as risk factors for melanoma (Tomao et al. 2014). Another proposed, but untested, pathway is the agonistic effect of CC on estrogen receptors in melanocytes. In vitro experiments with transfected cells have shown that CC exerts partial agonistic activity on estrogen receptors alpha (ERα) and beta (ERβ), corresponding to 12% to 25% of the activity of estradiol (Kuiper et al. 1997).

Estrogen signaling has been described in melanocytes and melanoma cells, although its role remains complex and context‐dependent. Among the receptor subtypes, ERβ is the most consistently reported in melanoma and has been associated with antiproliferative and potentially protective effects, whereas ERα is generally absent or expressed at low levels. These observations suggest that estrogen receptor–mediated pathways may influence melanoma biology, although their functional relevance is not yet fully understood (Marzagalli et al. 2015).

To date, no experimental study has evaluated the direct effects of CC on melanoma cells, leaving an important knowledge gap in understanding the safety profile of this widely used ovulation inducer. Therefore, the present study aimed to investigate the effects of CC on melanoma cell viability and metabolism, cell cycle progression, migratory capacity, and the modulation of genes involved in epithelial–mesenchymal transition and mitochondrial oxidative stress.

## 2 Methodology

### 2.1 Cell Culture and Viability Assay

The human melanoma cell line A375 was selected as an experimental model due to its well‐characterized molecular profile and relevance in studies of melanoma biology and pharmacological response (Hussein et al. 2025; Adu‐Amankwaah et al. 2025). Derived from a primary cutaneous melanoma of a 54‐year‐old female patient, A375 cells harbor the BRAF V600E mutation, a key driver of melanoma progression. Importantly, these cells express estrogen‐related receptors, particularly ERβ and G protein‐coupled estrogen receptor (GPER), while ERα is typically absent or minimally expressed (Marzagalli et al. 2015). ERβ has been associated with antiproliferative and protective roles in melanoma (Caruntu et al. 2016), highlighting the biological relevance of estrogen signaling in this model. In this context, the use of A375 provides a suitable platform to investigate the potential receptor‐mediated effects of CC, a selective estrogen receptor modulator.

The human melanoma cell line A375 was obtained from American Type Culture Collection and grown in DMEM supplemented with 10% of fetal bovine serum and 1% of penicillin–streptomycin (Sigma‐Aldrich Co., St. Louis, USA). Cells were plated at a density of 7500 cells per well and incubated for 24 h to allow proper adhesion and monolayer formation. After 24 h, when cells reached 70%–80% confluence, they were treated with four different concentrations of CC (2, 20, 200, and 2000 ng/mL) (Sigma) in serum‐free medium for 24 or 48 h. The CC concentrations were selected based on preliminary dose–response assays and in vitro pharmacokinetic estimates simulating clinical exposure ranges (Mikkelson et al. 1986).

Cell viability was assessed by measuring the rate of mitochondrial reduction of the yellow tetrazolium salt MTT (3‐(4,5‐dimethylthiazol‐2‐yl)‐2,5‐diphenyltetrazolium bromide; Sigma‐Aldrich, St. Louis, MO, USA) to insoluble purple formazan crystals. All analyses were performed in triplicate. To minimize variability associated with the MTT assay, all experiments were conducted using the same seeding density, reagent concentration, incubation time, and spectral reading parameters.

### 2.2 Analysis of Cell Cycle by Propidium Iodide (PI) DNA Staining Flow Cytometry

Cell cycle distribution and quantification of DNA fragmentation (hypodiploid DNA content) were assessed by PI staining (Nicoletti et al. 1991). A375 cells were seeded in 12‐well culture plates at a density of 70,000 cells per well, treated with CC (200 or 2000 ng/mL), and incubated in a humidified atmosphere containing 5% CO_2_ at 37°C for 24 or 48 h. Control cells were incubated with DMEM only. After treatment, the supernatant was collected. Cells were centrifuged and resuspended in hypotonic fluorochrome solution (HFS; 50‐μg/mL PI in 0.1% sodium citrate plus 0.1% Triton X‐100). Adherent cells were harvested for each treatment condition by trypsinization (Sigma‐Aldrich Co., St. Louis, MO, USA), centrifuged, washed with sterile PBS and resuspended in 300 μL of HFS. Cells were incubated in HFS at 4°C for 1 h in the dark. PI fluorescence from 10,000 individual nuclei was measured using a FACScan flow cytometer (BD Biosciences, USA) with FACSDiva software (BD Biosciences, USA). Data were analyzed using FlowJo software version 7.5.5 (Tree Star Inc., CA, USA). All groups were analyzed in triplicate in at least three independent experiments.

### 2.3 Cell Migration by Scratch Assay

Cells were seeded in 24‐well culture plates (Sarstedt AG & Co., Nümbrecht, Germany) at a density of 170,000 cells per well to reach 90% confluence after 24 h of incubation. Monolayers were scratched once using sterile 200‐μL micropipette tips to create a cell‐free gap (wound area) at the bottom of each well (Liang et al. 2007). The medium was aspirated, and wells were washed with sterile PBS to remove detached cells. Cells were then treated with CC (CC1: 200 ng/mL; CC2: 2000 ng/mL) diluted in DMEM. Control cells received DMEM only. Wounds were photographed at 0, 2, 24, and 48 h using an inverted microscope with a 4× objective and a 16‐megapixel camera (ASUS Zenfone 3 Max). Wound gaps were measured at two distinct points per image using ImageJ software (National Institutes of Health, USA), and mean wound width was calculated.

### 2.4 Regulatory Gene Expression Assays

Cells were seeded in 12‐well culture plates at 70,000 cells per well and incubated for 24 h prior to treatment. Cells were then treated with CC (200 or 2000 ng/mL) diluted in DMEM and incubated for 24 h. Control cells received DMEM only. After treatment, supernatants were transferred to microtubes and centrifuged to recover floating cells. Adherent cells were incubated for 3 min with 500 μL of TRIzol reagent (Invitrogen Life Technologies, Carlsbad, CA, USA). The contents of each well were transferred to microtubes for subsequent RNA extraction and quantification.

cDNA was synthesized from 1 μg of RNA using random hexamers (Invitrogen Life Technologies, Carlsbad, CA, USA). Quantitative PCR (qPCR) was performed using 50 ng of cDNA per reaction in a QuantStudio 3 Real‐Time PCR System. Primers were designed to hybridize to unique sequences of genes involved in epithelial–mesenchymal transition (E‐cadherin/CDH1 and N‐cadherin/CDH2), mitochondrial oxidative stress (SOD2), and oxidative stress–induced apoptosis (BAX). Each condition was analyzed in triplicate. Relative gene expression was calculated using the 2^−ΔΔCT^ method, with S26 as the housekeeping gene for normalization.

### 2.5 Statistical Analysis

After confirming normal distribution using the D'Agostino–Pearson test, group means were compared by one‐way ANOVA followed by Tukey's multiple comparison test.

## 3 Results and Discussion

**FIGURE 1:**
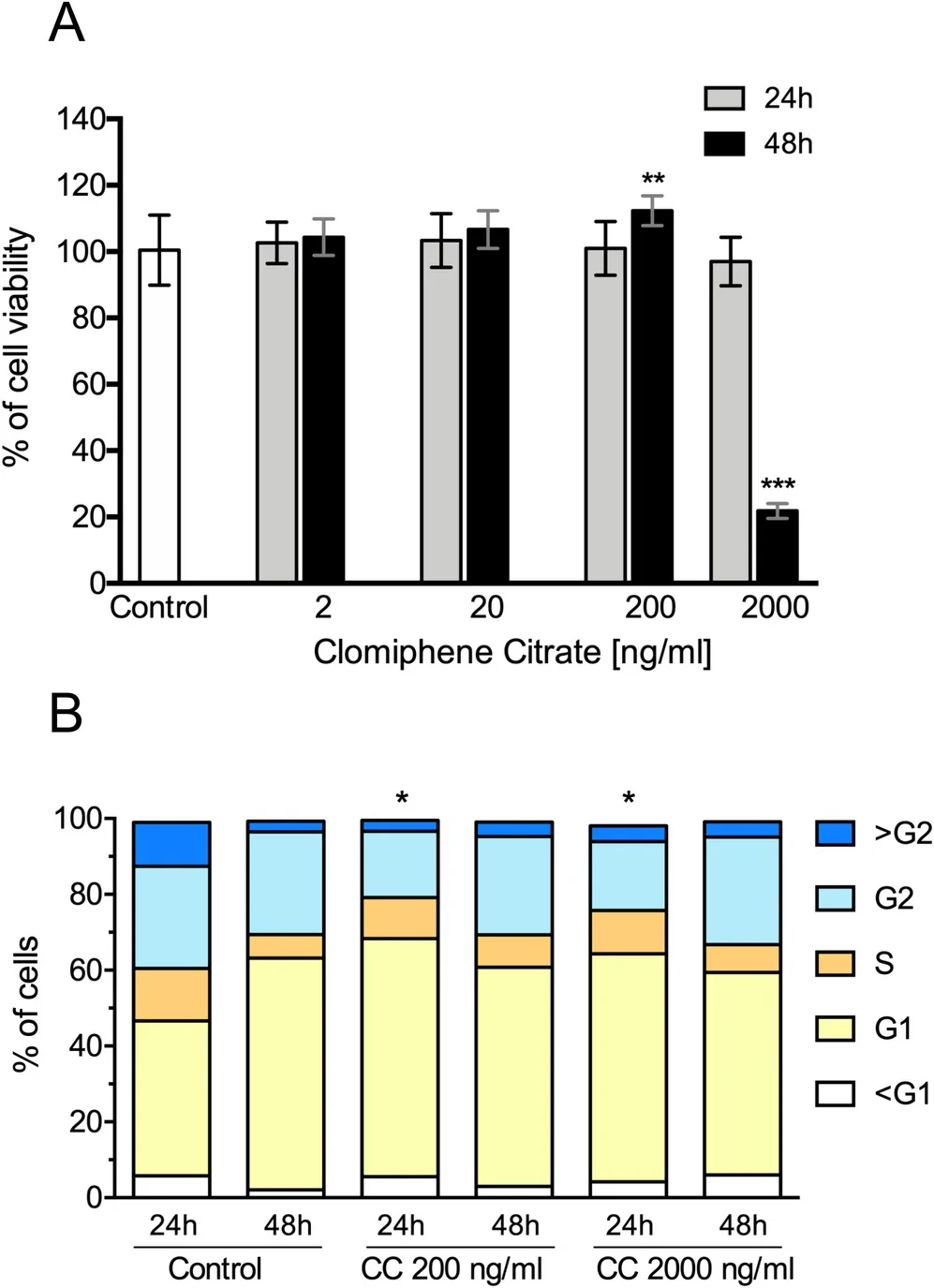
Cell viability assessed by MTT assay (A) and cell cycle phases (B) following 24‐ and 48‐h treatments with four concentrations of clomiphene citrate (CC) or only culture medium. Data are expressed as means ± SD. *p < 0.05; **p < 0.01; ***p < 0.0001 vs. control.

CC had no cytotoxic effects on MTT metabolism and cell viability during the first 24 h of incubation. However, 48 h after exposure to CC at the highest dose (2000 ng/mL), there was a significant decrease in cell metabolism compared to control (*p* < 0.0001, Figure 1A). These data provide evidence that the effects of CC on A375 cell viability and metabolism vary according to exposure time and drug concentration. MTT reduction directly depends on the activity of dehydrogenases, which are mitochondrial and cytoplasmic enzymes (Mosmann 1983). Thus, it is possible to infer that CC contributes to this enzyme's depletion and cytotoxicity only at high concentrations and long exposure times.

After 24 h of incubation, CC induced a significant increase (*p* < 0.001) in the percentage of cells in the G1 cell cycle phase in comparison to control cells (Figure 1B). At the same time, the rate of cells in S and G2 phases decreased. After 48 h of incubation, while the percentage of G1 cells grew in the control group, it slightly diminished in the CC‐treated groups (*p* < 0.001, Figure 1B). Cell cycle changes are one of the mechanisms through which melanoma cells self‐control their proliferation rate, and these changes are intimately related to cancer progression. CC treatment led primarily to a cycle arrest at the G1 phase, probably at the first cell cycle checkpoint before DNA duplication. This was observed after 24 h of incubation with both CC doses, indicating that cells paused their proliferation at an early interphase stage. Following 48 h of CC treatment, the cells presented a DNA fragmentation, with DNA content under the G1 phase (subdiploid), suggesting cell death associated with DNA fragmentation (Kim et al. 2015). While an increase in subdiploid DNA content is commonly associated with apoptosis, it is not specific to this process. In in vitro systems, DNA fragmentation may also occur in other forms of cell death, including necrosis, secondary necrosis, and regulated cell death (RCD) pathways such as necroptosis or parthanatos, particularly under conditions of cellular stress (Galluzzi et al. 2018). Therefore, the observed subdiploid population should be interpreted as evidence of cell death rather than definitive proof of apoptosis. Further studies using specific assays will be necessary to determine the type of cell death involved and to better understand the mechanisms underlying these effects.

**FIGURE 2:**
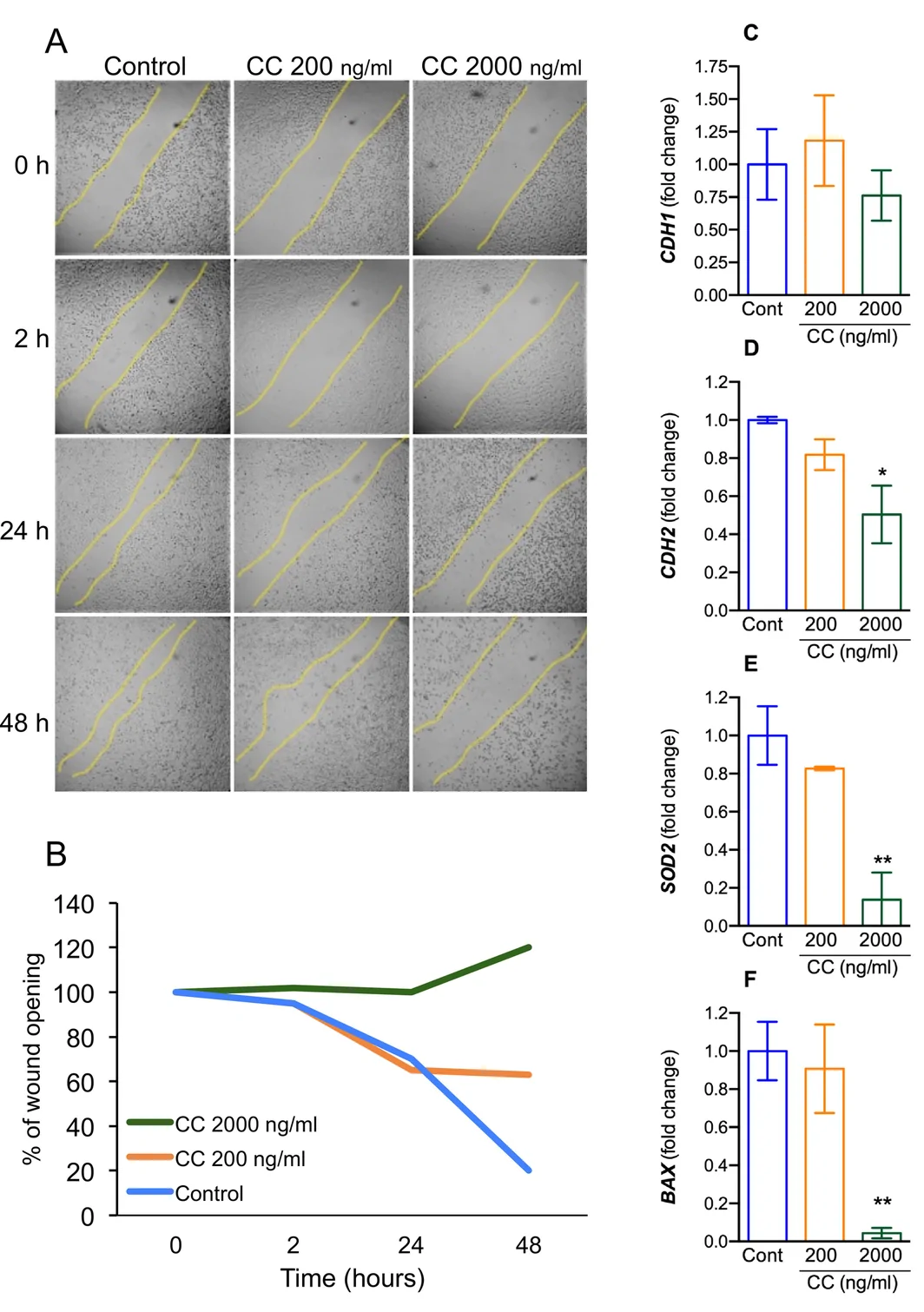
(A, B) Scratch assay showing the effects of clomiphene citrate (CC) on cell migratory potential over time. The yellow lines highlight the edges of the gap. (C–F) Expression of mRNA for the target genes *CDH1* (C), *CDH2* (D), *SOD2* (E), and *BAX* (F) in melanoma cells following 24‐h treatment with CC. Data are expressed as means ± SE. *p < 0.05; **p < 0.01 vs. control.

Cells treated with 200‐ng/mL CC retained migration and proliferation in the first 24 h but, after 48 h, the wound gap remained wider in the CC‐treated cells than in the control group (Figure 2A,B). As for the 2000‐ng/mL CC treatment, cell migration and proliferation were blocked, and the wound gap expanded following the initial monolayer scratch (Figure 2A,B). Thus, cell migration ability was compromised from the start, leading to inefficient gap closure and wound widening after 48 h of incubation with CC. Correlating these changes to cell cycle and viability suggests that the CC effects intensified after longer exposure, and drug concentration was also a key feature in the observed impacts.

The relative gene expression of *CDH2* was significantly reduced by CC at the higher dose (*p* < 0.05), whereas *CDH1* was unchanged (Figure 2C,D), suggesting that CC inhibited the epithelial–mesenchymal transition that confers a more invasive and metastatic phenotype to melanoma cells. N‐cadherin expression gives the cells a mesenchymal phenotype known for its greater motility and ability to navigate easily throughout the tissue, leading to a higher risk of metastasis (Loh et al. 2019). Considering the known metastatic potential of the A375 cell line, *CDH2* downregulation by CC suggests a protective mechanism activated by the drug. In addition, the *SOD2* gene that encodes the antioxidant enzyme superoxide dismutase 2 was downregulated after CC treatment (*p* < 0.01, Figure 2E), while the proapoptotic gene *BAX* was also downregulated by CC (*p* < 0.01, Figure 2F). The decrease in *SOD2* expression impairs cell protection against oxidative stress, suggesting that CC‐treated cells could have become more susceptible to oxidative damage. Oxidative stress can induce apoptosis through BAX signaling pathways (Oyagbemi et al. 2019). Thus, *BAX* inhibition after CC treatment suggests a cell response to the mitochondrial oxidative stress, preventing apoptosis from being initiated through the BAX mitochondrial signaling pathway.

A strength of this study is the use of multiple methods to comprehensively assess the direct effects of CC on melanoma cells. Further strengths are the use of clinically representative drug concentrations and the well‐controlled experimental conditions. Nevertheless, it has some limitations. Our data are restricted to a well‐characterized epithelial cell line isolated from the skin of a patient with malignant melanoma. Thus, the use of a single melanoma cell line may limit the generalizability of the findings, and further studies using additional melanoma cell lines with distinct molecular profiles, as well as normal melanocytes, are warranted to confirm the broader applicability of our results. However, it should be noted that the use of primary human melanocytes as in vitro controls presents important limitations, including limited lifespan in culture and the lack of well‐defined donor characteristics such as age, sex, and anatomical origin, which may influence cellular responses and limit direct comparison with melanoma models derived from adult female patients (Goff et al. 2023; Kohli et al. 2017). In addition, further studies in animal models that replicate the systemic conditions involved in melanoma initiation and progression are warranted. Our findings do not prove that CC effects were mediated by its weak agonistic binding to ER, although this is the most plausible mechanism (Kuiper et al. 1997). Moreover, our data do not allow conclusions regarding the involvement of ER–mediated pathways, as this would require dedicated experimental approaches, such as receptor expression analysis and functional modulation strategies. Therefore, further studies specifically designed to address this question are warranted. Finally, we cannot rule out the possibility that CC affects the risk of melanoma indirectly by modulating pituitary or ovarian hormones.

In conclusion, the overall results of the present study indicate that CC treatment does not increase the malignant potential of human melanoma cells in vitro but may even decrease some of their malignant features. These findings reassure the safety of CC, which has been documented over decades of use by millions of patients worldwide.

## Funding

This research was supported by the Conselho Nacional de Desenvolvimento Científico e Tecnológico (CNPq) and Fundação de Amparo à Pesquisa do Estado de Minas Gerais (FAPEMIG, grants # APQ‐02202‐21 and APQ‐01247‐23).

## Disclosure

The authors have nothing to report.

## Data Availability

The data underlying this article will be shared on reasonable request to the corresponding author.
